# IL-1β and HMGB1 in Epileptogenesis: Recent Advances and Clinical Translation

**DOI:** 10.3390/ph18101522

**Published:** 2025-10-10

**Authors:** Huali Geng, Leihao Sha, Lei Chen

**Affiliations:** 1 Department of Neurology, West China Hospital, Sichuan University, Chengdu 610041, China; ml465463@163.com (H.G.);; 2 High Altitude Medicine Key Laboratory of Sichuan Province, West China Hospital, Sichuan University, Chengdu 610041, China

**Keywords:** epilepsy, IL-1β, IL-1R, HMGB1, TLR4, therapeutic targets, clinical translation

## Abstract

Epilepsy is one of the most prevalent and disabling neurological disorders, affecting approximately one percent of the population. Due to the complex pathophysiology underlying drug-resistant epilepsy, nearly one-third of patients with epilepsy do not benefit from current treatments. Neuroinflammation is one of the most well-studied pathways in epileptogenesis, and inflammatory mediators play a crucial role in this process. The IL-1β/IL-1R1/IL-1Ra and HMGB1/TLR4 pathways play significant roles in epileptogenesis in both animal and human studies. Interventional investigations on the IL-1β/IL-1R1/IL-1Ra and HMGB1/TLR4 pathways showed antiseizure effects, suggesting that these pathways could be therapeutic targets for epilepsy. However, related targeted treatments are limited in clinical practice. In this work, we evaluated the advances of the IL-1β/IL-1R1/IL-1Ra and HMGB1/TLR4 pathways in epileptogenesis, as well as clinical trials or interventional investigations of current medications or substances targeting these pathways. To facilitate clinical translation, we highlighted the gap between research advancements and clinical practice and presented several strategies for closing the gap to fulfill the urgent requirements of patients with epilepsy.

## 1. Introduction

Epilepsy is one of the most common chronic neurological diseases, affecting approximately 1.0% of the world’s population, about 65 million people worldwide [1,2]. Unpredicted seizures bring a great burden to patients with epilepsy and could be lethal due to sudden unexpected death in epilepsy, status epilepticus, and seizure-related accidents (for example, drowning, fall, or vehicle accidents) [3,4]. Epileptogenesis refers to the process by which a normal brain becomes epileptic [5], which is associated with multiple mechanisms, including transcriptomic and epigenetic modifications [6,7], altered molecular pathways [5,8], and blood–brain barrier dysfunction [9].

This involves multiple mechanisms, including transcriptomic and epigenetic modifications, altered molecular pathways, and blood-brain barrier dysfunction. The mechanisms leading to epilepsy are complex, and as a result, approximately 30% of patients develop drug-resistant epilepsy and cannot benefit from existing treatments [10,11]. Understanding the pathogenic processes of epileptogenesis and developing tailored interventions may help prevent epileptic seizures and reduce seizure burden, which is desperately needed in practice.

The accumulation of evidence has led to the proposal of mechanisms of neuroinflammation in epilepsy [12,13,14]. Neuroinflammation in the central nervous system (CNS), or neuroinflammation, is frequently activated in epileptogenic brain regions in humans and is clearly present in animal models of epileptogenesis and chronic epilepsy, which suggests that it plays a crucial role in both the development (epileptogenesis) and maintenance of the disease (epilepsy) [15,16,17]. Neuroinflammation can be caused by numerous deleterious stimuli, including trauma, stroke, exposure to toxic substances, infection or stress experienced and mediated by the synthesis of cytokines, chemokines, complement factors and other inflammatory molecules [1,14], which are mainly produced and released by brain cells (i.e., microglia, astrocytes, and neurons), endothelial cells of the blood–brain barrier (BBB) and peripheral immune cells [1,18]. Among them, Interleukin-1beta (IL-1β), and High mobility group protein B1 (HMGB1) and their related pathways demonstrated an essential role in epileptogenesis and potential as therapeutic targets, which have been given much focus in the research field [19,20,21]. In this review, we mainly focus on the IL-1β/IL-1R1/IL-1Ra pathway and HMGB1/TLR4 pathway, in which recent advances in knowledge have been made for epilepsy. We summarize evidence from animal models and clinical studies, aiming to exemplify how such dysregulated neuroinflammatory signals can contribute to epileptogenesis and their potential as therapeutic targets. Towards clinical translation, we carefully reviewed existing substances and drugs targeting the IL-1β/IL-1R1/IL-1Ra pathway and HMGB1/TLR4 pathway and their effects on epilepsy models and patients. Based on existing evidence, we further propose potential ways to bridge the gap between mechanisms and clinical practice.

## 2. IL-1β/IL-1R1/IL-1Ra and Epilepsy

### 2.1. IL-1β/IL-1R1/IL-1Ra Pathway

Interleukin-1 (IL-1) serves as a pivotal inflammatory cytokine central to innate immunity and inflammatory responses. Its two principal members, Interleukin–1 (IL-1α) and IL-1β, signal through a common receptor complex yet exhibit distinct biological characteristics: IL-1α is constitutively expressed and released upon cellular damage to mediate sterile inflammation, whereas IL-1β, a major proinflammatory regulator, is synthesized as an inactive precursor (pro-IL-1β) whose activation strictly depends on caspase-1-mediated proteolytic cleavage within the inflammasome—a mechanism particularly crucial in neuroinflammatory disorders [22,23]. Both ligands initiate signaling by binding to the type I IL-1 receptor (IL-1R1), leading to the recruitment of the coreceptor IL-1 receptor accessory protein (IL-1RAcP) to form a high-affinity signaling complex. This pathway is tightly modulated by the endogenous antagonist IL-1 receptor antagonist (IL-1Ra), which competitively binds to IL-1R1 without enabling IL-1RAcP recruitment, thereby effectively inhibiting downstream signaling [24]. Thus, the inflammation activated by the IL-1β/IL-1R1/IL-1Ra pathway is under precise regulation. In non-inflammatory states, IL-1β is present at a low level in the peripheral blood and in the CNS, which mainly participates in the processes of sleep, learning, memorization, and neuromodulation [1,14]. Microglia, astrocytes, neurons, endothelial cells of the BBB, and leukocytes extravasated into the brain are the cell sources to produce IL-1β in the brain, and these cells also express a basal level of IL-1R1. Therefore, IL-1β pathway could act via autocrine and paracrine messaging [14].

### 2.2. IL-1β/IL-1R1/IL-1Ra in Patients with Epilepsy

A growing number of studies have revealed altered levels of IL-1β in seizures and epilepsy. The majority of them indicate IL-1β concentration. Upregulation of IL-1β can be detected in the epileptogenic cortex in various types of epilepsy in patients and animal models [25,26,27,28]. These findings suggested the probable close link between IL-1β signal and the pathophysiology of epilepsy, which is further confirmed by the evidence that no IL-1β- and IL-R1-immunoreactive neurons were observed in the hippocampus of patients with extrahippocampal epileptogenic lesions [28]. In addition, the investigation of IL-1β- and IL-1R1- immunoreactivity in temporal lobe epilepsy with hippocampal sclerosis brain specimens supported the correlation of IL-1β signal to neuronal cell loss and BBB permeability alteration [28].

IL-1β can also be detected in extra-brain samples from patients with seizures and epilepsy, such as serum, plasma, cerebrospinal fluid (CSF), especially in serum specimens. Most studies suggested serum IL-1β was significantly elevated in epileptic patients [26,29,30,31,32,33], which indicated serum IL-1β as a biomarker for epilepsy. Kamaşak et al. observed that serum IL-1β levels in the severe epilepsy group were higher than in the mild epilepsy group and the control group, and were higher in the mild epilepsy group than in the control group, while IL-1R1 was higher in the severe epilepsy group than in the control group [33]. Lorigados Pedre et al. reported a statistically significant decrease in IL-1β in the serum of patients with DRTLE one and two years after surgery. Interestingly, one year after surgery treatment, lower serum levels of IL-1β were observed in seizure-free patients, while patients who remained with seizures showed higher serum levels of IL-1β [26]. These established evidences highlighted the correlation of serum IL-1β with severity and drug resistance in patients with epilepsy, though more investigations considering various etiologies of epilepsy are required.

Upregulation of IL-1β in brain tissue, CSF, and serum in patients with epilepsy, together with evidence that serum concentration of IL-1β decreased once the epileptogenic zone was resected and seizure activity was reduced [26], drives a potential hypothesis that CNS IL-1β is associated with serum IL-1β in epileptic patients. This is supported by the involved role of IL-1β in BBB permeability alteration and a significant increase in CSF-serum ratio of IL-1β in patients with epilepsy [28,34].

As an endogenous antagonist of IL-1β signal, IL-1Ra was shown to be elevated in serum or in CSF from patients with epilepsy [29,32]. However, reduced expression of intracellular IL-1Ra isoforms and a functional deficiency in IL-1Ra inhibitory activity were indicated [29]. Further, a significant decrease in IL-1Ra/IL-1β ratio suggested insufficiency of endogenous inhibitory activity and upregulation of IL-1β signal in epilepsy [34].

### 2.3. Mechanisms of IL-1β/IL-1R1/IL-1Ra Pathway in Epilepsy

**Figure 1:**
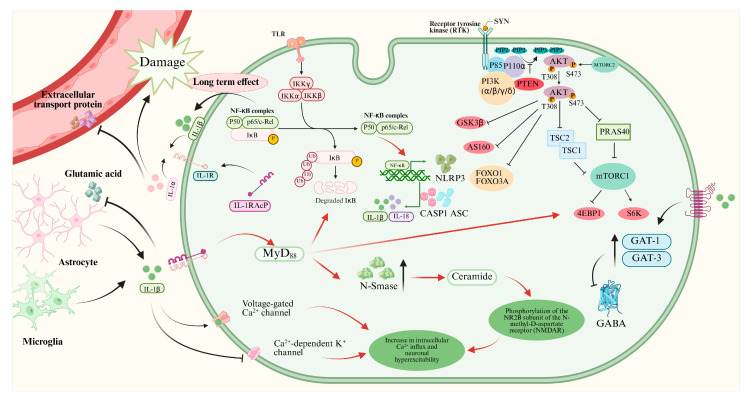
Mechanisms of IL-1β/IL-1R1/IL-1Ra pathway in neurons of epileptogenesis. This figure illustrates how IL-1β binding to IL-1R1 activates MyD88-dependent signaling pathways (e.g., PI3K/Akt/mTOR and NF-κB), leading to neuroinflammation, increased synaptophysin expression, enhanced NMDA receptor function, reduced GABAergic inhibition, and blood–brain barrier disruption, collectively contributing to neuronal hyperexcitability and epileptogenesis.

In the past decades, many studies reported that IL-1β could promote epileptogenesis by various mechanisms (Figure 1). However, the dominating mechanism is still unknown. IL-1β is produced and released mainly by microglia and astrocytes in the brain, and possibly leukocytes extravasated from the peripheral blood circulation through the altered blood–brain barrier [14]. IL-1β in the extracellular space will bind with IL-1R1, which leads to the activation of myeloid differentiation factor 88 (MyD88). PI3K/Akt/mTOR signaling and NF-κB signaling are activated by MyD88 [14]. The expression of synaptophysin (SYN) will be upregulated via the PI3K/Akt/mTOR signaling during epileptogenesis, which results in Ca^2+^-dependent release of neurotransmitters [35]. Moreover, the translocation of nuclear factors κB (NF-κB) is increased by activation of IL-1β/IL-1R1/IL-1Ra pathway [36]. The NF-κB signaling promotes transcription of inflammatory genes, including NOD-like receptor protein 3 (NLRP3), which leads to production of more IL-1β and IL-18 with the help of caspase 1 (CASP1) and apoptosis speck-like protein (ASC) [37,38]. The long-term effect of NF-κB signaling will aggravate the process of neuroinflammation.

The binding of IL-1β and IL-1R1 will lead to upregulation of neutral sphingomyelinase (N-Smase), which promotes the production of ceramide. Ceramide accelerates the protein-tyrosine kinase (Src-kinase)-mediated phosphorylation the of the NR2B subunit of the N-methyl-D-aspartic acid receptor (NMDAR), which leads to increased Ca^2+^ influx and neuron hyperexcitability [39,40,41]. Moreover, IL-1β can promote Ca^2+^ influx by activating voltage-gated Ca^2+^ channels and inhibiting Ca^2+^-dependent K^+^ channels directly [12,42].

Furthermore, IL-1β can decrease the concentration of inhibitory neurotransmitter gamma-aminobutyric acid (GABA) by increasing the expressions of GABA transporter type 1 and type 3 (GAT-1 and GAT-3), which results in neuron hyperexcitability and ictogenesis [43,44]. Moreover, IL-1β can inhibit the glutamate reuptake by astrocytes, which will aggravate the imbalance of Excitatory and inhibitory transmitters [14].

IL-1β also affects the blood–brain barrier in epilepsy. IL-1β can damage the tight junctions between endothelial cells of brain capillaries and inhibit the expression of efflux transporters, including p-glycoprotein and breast cancer resistance protein [45,46]. Moreover, IL-1β promotes the pericyte modifications and pericyte–microglia aggregation [14,47], which all result in damage to the blood–brain barrier. Circulating proinflammatory substances and leukocytes can penetrate the blood–brain barrier and aggravate the neuroinflammation.

### 2.4. Substances Targeting IL-1β/IL-1R1/IL-1Ra Pathway

Due to the importance of the IL-1β/IL-1R1/IL-1Ra pathway in epileptogenesis, the IL-1β/IL-1R1/IL-1Ra pathway is considered a potential target for epilepsy treatment. An increasing number of drugs targeting this pathway are being developed (Table 1). VX-765 is a reversible caspase-1 inhibitor that blocks IL-1β maturation and suppresses IL-1β/IL-1R1 signaling, a pathway critically involved in drug-resistant epilepsy via neuroinflammation and blood–brain barrier disruption. In a phase IIa randomized controlled trial involving 60 patients with drug-resistant focal epilepsy, VX-765 reduced seizure frequency by 15.6% (vs. 7.0% placebo) over 6 weeks without statistical significance, but post hoc analysis showed a 50% responder rate of 31.3% (vs. 8.3% placebo) during the last 4-week period, suggesting delayed effects [48]. Treatment-related adverse events occurred in 72.9% of patients, with 6.3% experiencing serious events. The clinical Phase IIb trial of VX09-765 was discontinued for business reasons. Anakinra, an IL-1 receptor antagonist, pharmacologically inhibits the IL-1β/IL-1R1/IL-1Ra pathway. Although it has not been tested in prospective clinical trials in patients with epilepsy, there are many case reports demonstrating its efficacy in adults and children with Febrile infection-related epilepsy syndrome (FIRES) [49,50], adults with super-refractory status epilepticus [51], and children with drug-resistant epilepsy [52]. In addition, Lai et al. used anakinra, an IL-1 receptor antagonist, in 25 children with FIRES to block IL-1β signaling and mitigate neuroinflammation. Earlier treatment correlated with shorter duration of mechanical ventilation, ICU and hospital stay, but risks included infection, transaminitis, and cytopenia [53]. GAOMA Therapeutics’ novel anti-seizure drug, GAO-3-02, currently under development, has the potential to target the IL-1β/IL-1R1/IL-1Ra pathway and has also been found to inhibit seizures and improve cognition in preclinical experiments [54].

In addition to drugs already in preclinical and clinical trials, many studies have reported substances with therapeutic effects that target the IL-1β/IL-1R1/IL-1Ra pathway (Table 2). IL-1β-mediated PI3K/AKT/mTOR signaling pathway hyperactivation is associated with increased release of neurotransmitters and neuron hyperexcitability. Mycophenolate mofetil, LncRNA MEG3, saikosaponin, and biochanin A had a targeted effect on this pathophysiological mechanism [55,56,57,58]. Inhibiting IL-1β-mediated PI3K/AKT/mTOR signaling is associated with reduced seizures and seizure-related neuron injury in such experiments. Targeting IL-1β-mediated NF-κB signaling is also a therapeutic choice for epilepsy-related neuroinflammation. Rhein, phyllathin, and chondroitin sulfate can inhibit IL-1β-mediated NF-κB signaling to achieve antiseizure effects [59,60,61]. Inhibiting IL-1β-mediated NF-κB signaling is associated with decreased proinflammatory cytokines, and delayed onset and decreased severity of seizures. Biochanin A can inhibit the NLRP3 and its associated inflammasome, which is also associated with decreased production of IL-1β and other proinflammatory cytokines. Gimenes et al. reported that ANXA1-derived peptide Ac2-26 was associated with reduced neuronal injury and inflammation related to status epilepticus by modulation of the levels of formyl peptide receptor 2 and phosphorylated extracellular signal-regulated kinase, which leads to inactivation of astrocytes and decreased production of IL-1β [62]. However, most evidence remains preclinical, with limited human data on efficacy and safety. Potential risks include immunosuppression, off-target effects, and hepatorenal toxicity, highlighting the need for further clinical validation.

From the evidence above, we find many substances that have been successfully developed for many years as potential new options for targeting neuroinflammatory responses in epilepsy treatment. Anakinra was approved for marketing by the FDA in 2001 and is now used to treat rheumatoid arthritis, neonatal-onset multisystem inflammatory disease, and deficiency of interleukin-1 receptor antagonist [63,64]. Mycophenolate mofetil was approved for marketing by the FDA in 1995 and is now used to prevent the rejection of kidney, heart, or liver transplants [65]. Chondroitin sulfate is an OTC dietary supplement in North America, and it is a prescription drug in Europe to alleviate pain and inflammation from primary osteoarthritis [66]. The above evidence suggests that drugs used to treat peripheral inflammation may have potential anti-central nervous system inflammatory effects. Central nervous system drugs require the ability to cross the blood–brain barrier. We further discuss in “Potential of nano drug delivery system in treatment” how to improve the blood–brain barrier permeability of already existing drugs for peripheral inflammation, so that they could be reused in central nervous system inflammation.

## 3. HMGB1/TLR4 and Epilepsy

### 3.1. HMGB1/TLR4 Pathway

Toll-like receptors (TLRs) recognize various microbial pathogen molecules called pathogen-associated molecular patterns (PAMPs) [67]. Among them, TLR4 is one of the TLR family receptors, which is mainly expressed in the brain [68]. Its main ligand is lipopolysaccharide (LPS) from Gram-Negative bacteria. TLR signaling can be activated by the endogenous molecules of the damaged problem, which are called damage-associated molecular patterns (DAMPs). HMGB1, a ligand of TLR4 that participates in chromatin regulation in a physiological state, is an intranuclear non-histone protein that can be actively or passively released from damaged cells [69]. In the event of cell injury or stress, extracellular HMGB1 is partially oxidized to HMGB1 disulfide, which is recognized by TLR4 and advanced glycation end product (RAGE) receptors on the surface of neurons and glial cells, and it exerts a proinflammatory mediator function [70,71]. HMGB1/TLR4 signaling activation exacerbates neuroinflammatory responses and is involved in the pathogenesis of various neurological diseases, so HMGB1 is regarded as a key alarmin molecule connecting innate immunity and neuroinflammation.

### 3.2. HMGB1/TLR4 Pathway in Patients with Epilepsy

Many studies have reported increased levels of HMGB1 in patients with epilepsy. Upregulation of HMGB1 can be detected in the olfactory bulbs of patients with frontal lobe epilepsy [72], anterior hippocampal samples of patients with temporal lobe epilepsy (with and without hippocampal sclerosis) [73], and cerebrospinal fluid of patients with febrile seizures [74]. These results indicate that the upregulation of HMGB1/TLR4 is common in the brains of patients with epilepsy and suggest its ongoing role in the pathophysiology of the disease.

Upregulation of HMGB1 could also be detected outside of the brain. Patients with epilepsy demonstrated increased levels of HMGB1 (by nearly 80%) in the serum after generalized convulsive seizures [75]. And similar results were observed in children with febrile seizures [30]. During intervals of seizures, increased levels of HMGB1 were also detected in the serum of patients with epilepsy [76]. Such evidence indicated that HMGB1 could be a biomarker of epilepsy. Zhu et al. reported that serum concentration of HMGB1 could be a predictor of epilepsy prognosis in children with epilepsy, establishing a sensitivity of 80.6% and specificity of 92.5% [77]. Concentrations of HMGB1 are also correlated with increased risk and severity of epilepsy in adults [78]. Moreover, concentrations of HMGB1 could be predictors of drug treatment response in patients with epilepsy. Walker et al. reported a total area under the curve of 0.99 when using serum concentrations of HMGB1 to separate patients with drug-resistant epilepsy from patients with drug-responsive epilepsy and healthy controls [79]. These results demonstrated the vital role of the HMGB1/TLR4 pathway in patients with epilepsy and its potential for clinical translation.

**Figure 2:**
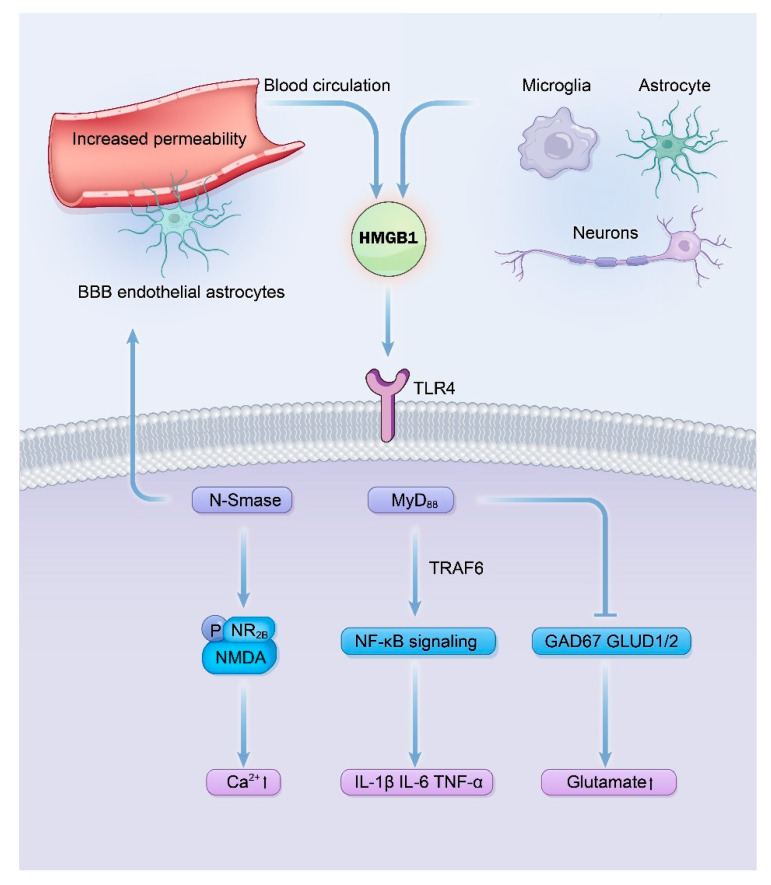
Signaling mechanisms of the HMGB1/TLR4 pathway in driving epileptogenesis. This diagram delineates the pro-epileptogenic cascade initiated by the binding of HMGB1, released from activated glial cells or neurons, to TLR4. Key consequences include the recruitment of myeloid differentiation primary response 88 (MyD88), leading to Nuclear Factor kappa B (NF-κB) activation and sustained neuroinflammation via Proinflammatory cytokine production (e.g., IL-1β); enhanced N-Methyl-D-Aspartic acid (NMDA) receptor function through a ceramide-dependent pathway, increasing neuronal Ca^2+^ influx and excitability; and a shift in neurotransmission balance towards excitation by impairing gamma-aminobutyric acid (GABA) synthesis. These interconnected mechanisms collectively underlie neuronal hyperexcitability and seizure generation.

HMGB1 is primarily produced and released by microglia, astrocytes, and neurons in the brain, and leaked from the peripheral blood circulation through the altered blood–brain barrier [40]. Figure 2 shows the mechanisms of the HMGB1/TLR4 pathway in epilepsy. Because the IL-1R1 and TLR4 shared the same intracellular domains, the mechanisms of HMGB1/TLR4 pathway in epilepsy are very similar to those of IL-1β/IL-1R1/IL-1Ra pathway [14,17]. NF-κB signaling will be activated after the binding of HMGB1 and TLR4, which promotes the release of proinflammatory cytokines such as IL-1β and IL-6 [80,81]. HMGB1 can also affect the influx of Ca^2+^ by upregulation of N-Smase, which promotes phosphorylation of NR2B of the NMDA through ceramide [82,83]. HMGB1 can also lead to an imbalance of glutamate and GABA by down-regulated expression of glutamate decarboxylase67 (GAD67) and glutamate dehydrogenase (GLUD1/2) [83].

Furthermore, HMGB1 demonstrated similar damage effects to the blood–brain barrier as IL-1β. HMGB1 can directly bind to TLR4 in the endothelial cells and astrocytes of the blood–brain barrier and induces circling albumin to invade the microenvironment of the brain, which aggravates the neuroinflammation by activating astrocytes [84,85].

### 3.3. Substances Targeting HMGB1/TLR4 Pathway

Maroso et al. reveal for the first time that the HMGB1/TLR4 pathway is a potential target for epilepsy treatment [82]. Although no drugs targeting the HMGB1/TLR4 pathway for epilepsy are currently in clinical trials, many substances can target this pathway and thus exhibit anti-epileptic effects (Table 3). Anti-HMGB1 monoclonal antibody exhibits antiseizure effects in both mouse and zebrafish models of epilepsy and can improve epilepsy-related memory impairment [86,87]. Glycyrrhizin, a drug targeting HMGB1, reduces mortality and neurological injury of rats in a model of status epilepticus [88]. In addition, Celecoxib, an HMGB1 translocation inhibitor, demonstrated antiseizure effects in two studies [89,90]. Other drugs targeting the shared mechanism of the HMGB1/TLR4 pathway and the IL-1β/IL-1R1/IL-1Ra pathway have also shown significant antiepileptic effects, such as TAK-242 [91], Fisetin [92], and Resveratrol [93]. Similarly to many drugs targeting the IL-1β/IL-1R1/IL-1Ra pathway, anti-HMGB1 drugs used to treat peripheral inflammation have demonstrated a role in treating inflammatory responses in the central nervous system. Celecoxib was approved by FDA in 1998 and is currently used to treat osteoarthritis, rheumatoid arthritis, acute pain, menstrual symptoms, and to reduce polyps in familial adenomatous polyposis, which demonstrated antiseizure effects [94]. To date, most evidence derives from animal models, and clinical trials evaluating efficacy, optimal dosing, and safety in epilepsy patients are scarce. Potential disadvantages of these immunomodulatory approaches include immunosuppression, off-target effects, and species-specific differences in drug response. We further discuss how to reuse peripheral anti-inflammatory drugs in the central nervous system in the “Potential of nano drug delivery system in treatment” section.

## 4. Discussion

The current review highlights the roles of the IL-1/IL-1R1/IL-1Ra pathway and HMGB1/TLR4 pathway as crucial mechanisms underlying epileptogenesis and sustaining epilepsy, making them potential therapeutic targets. Anti-neuroinflammation therapy could be a potential option for adjuvant antiseizure treatment. However, there are currently no available drugs in clinical practice. Uncertain mechanisms could pose a barrier to clinical translation. Even though neuroinflammation in epilepsy has been reported to be linked to various mechanisms, the lack of knowledge on the priorities of these mechanisms makes it challenging to develop targeted drugs. How to reuse the existing anti-inflammatory drugs in the central nervous system could pose another barrier. Currently, there are many anti-inflammatory drugs that are being developed or in clinical trials that target the IL-1/IL-1R1/IL-1Ra pathway and the HMGB1/TLR4 pathway. For example, MK-7110, a novel human CD24 fusion protein developed by OncoImmune and MSD and reported to target HMGB1 and mediated pathways, is currently in clinical trials for Graft-vs-Host Disease [95]. Reusing existing drugs could save the huge resources required to develop a new drug. However, how to cross the blood–brain barrier must be solved, as most of them are traditionally indicated for peripheral inflammation. Here, we propose two possible ways to accelerate clinical translation.

### 4.1. Targeting the Shared Mechanisms

The HMGB1/TLR4 pathway and IL-1/IL-1R1/IL-1Ra pathway share the same intracellular domains of receptors, and these pathways have similar downstream mechanisms in neuroinflammation, such as increased Ca^2+^ influx of NMDA [96,97]. TLR4 and IL-1R1 share intracellular domains that can be targeted by drugs to inhibit these mechanisms in both pathways. These promising targets have the potential to be valuable in both drug development and clinical practice. Moreover, MyD88 played a crucial role in both pathways. Liu et al. reported that inhibition of MyD88 could provide neuroprotective effects after status epilepticus in mice [98]. In summary, the HMGB1/TLR4 pathway and IL-1/IL-1R1/IL-1Ra pathway partly share similar mechanisms in neuroinflammation of epilepsy. Epilepsy treatment may benefit from targeting the shared mechanisms of both pathways.

### 4.2. Potential of the Nano Drug Delivery System in Treatment

With recent development of nanotechnology, nanoparticles have been widely investigated in the field of biomedicine [99,100]. Nanoparticles ranged from 1 to 100 nm, which is similar to many biological molecules in scale. Therefore, nanoparticles could be designed to have many biological functions, including increased transport through the blood–brain barrier, which is an essential property for drugs targeting the central nervous system [101]. Moreover, many studies have developed nano-based antiseizure medication to achieve higher permeation of the blood–brain barrier and better treatment response. Wang et al. used alpha-methyl-L-tryptophan, an increased uptake amino acid by the blood–brain barrier in patients with epilepsy, to develop a novel nanoparticle, achieving higher concentration in epileptic foci in an acute temporal lobe epilepsy model [102]. Wu et al. reported another novel nanoparticle designed with angiopep-2, a 19-mer peptide with high affinity to the low-density lipoprotein receptor-related protein 1 on the blood–brain barrier, achieving higher concentrations of antiseizure medications and better seizure control in different animal models of epilepsy [103]. Such evidence indicated the potential of nanotechnology in the treatment of epilepsy.

It is worth mentioning that beyond the aforementioned nanodrug strategies, stem cell-derived tools such as mesenchymal stem cell exosomes (MSC-Exos) have shown promise in targeting neuroinflammation [104]. These exosomes can effectively deliver functional miRNAs (e.g., miR-129-5p) to suppress the HMGB1/TLR4 and IL-1 family signaling pathways, thereby alleviating neuroinflammation and modulating aberrant neurogenesis [105,106]. Studies have demonstrated that exosomes not only cross the blood–brain barrier efficiently but also serve as natural nanocarriers for anti-inflammatory molecules, reducing seizure severity and improving neurological outcomes in epilepsy models. Such extracellular vesicle-based delivery systems represent a novel therapeutic strategy with high potential for clinical translation in inflammation-related epilepsy.

The development of novel drugs is a time-consuming and resource-intensive task, and could take decades and billions of dollars. Current medications that target the IL-1/IL-1R1/IL-1Ra pathway and the HMGB1/TLR4 signaling pathway are primarily prescribed for peripheral inflammation disorders that have difficulty penetrating the blood–brain barrier. Combining nanotechnology and existing drugs targeting neuroinflammation pathways may be a promising approach to treating neuroinflammation in epilepsy due to the transport properties of nanoparticles. For this reason, future studies may benefit from nano-assisted anti-inflammatory therapy.

## Figures and Tables

**Table 1:** Drugs targeting IL-1β/IL-1R1/IL-1Ra pathway in epilepsy. The table summarizes drugs in preclinical or clinical development targeting the IL-1β pathway. VX-765 inhibits caspase-1 to reduce IL-1β maturation; Anakinra is an IL-1R antagonist repurposed for FIRES; GAO-3-02 is a novel anti-seizure drug with anti-inflammatory effects. Clinical outcomes include seizure reduction and cognitive improvement, but most agents remain investigational.

Drug	Structure	Target and Mechanisms	Latest Status	Outcome	Ref.
VX09-765	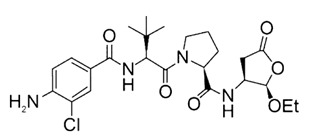	Caspase 1 IL-1β synthesis inhibitor	Clinical: Phase IIa for drug-resistant focal-onset epilepsy in adults (Phase IIb enrolled but stopped for business reasons)	A ≥50% reduction in seizures in 18.8% of subjects in the VX-765 group versus 8.3% in the placebo group (*p* > 0.05); 31.3% in the VX-765 group compared with 8.3% in the placebo group in the post hoc analysis, suggesting a delayed effect	[48]
Anakinra	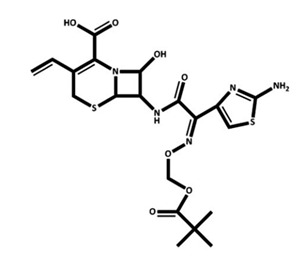	IL-1 receptor IL-1 receptor antagonist	Clinical: Retrospective cohort for febrile infection-related epilepsy syndrome (FIRES) in children case reports for FIRES (adult), super-refractory status epilepticus in autoimmune encephalitis in adults, and drug-resistant epilepsy in children	FIRES: Seizure reduction was associated with the use of anakinra in both children and adults. Super-refractory status epilepticus in autoimmune encephalitis: This patient made a recovery with effective communication and the ability to walk with assistance after 16 weeks of coma. Drug-resistant epilepsy: reduction in seizures and improvement of cognition.	[49,50,51,52,53]
GAO-3-02	N/A	None declared	Preclinical phase	Action on IL-1β: GAO-3-02 concentration-dependently resolved the inflammatory response induced by IL-1β in immortalized human microglial cells Action on epilepsy: reduced seizures and possible benefits on cognition	[54]

**Table 2:** Substances targeting IL-1β/IL-1R1/IL-1Ra pathway in epilepsy. This table lists natural and synthetic substances modulating IL-1β-related pathways in animal models. Mechanisms include inhibition of PI3K/Akt/mTOR or NF-κB signaling, NLRP3 inflammasome suppression, and neurotransmitter regulation. Outcomes indicate reduced seizure severity and neuroinflammation, but evidence is primarily preclinical.

Substance	Targeted Mechanism	Model	Outcome	Ref.
Mycophenolate mofetil	IL-1β-mediated PI3K/AKT/mTOR signaling pathway hyperactivation	Lithium pilocarpine induced recurrent seizure in rats	Decreased recurrent seizures and improved neurobehavioral comorbidities	[55]
LncRNA MEG3	IL-1β-mediated PI3K/AKT/mTOR signaling pathway hyperactivation	Lithium chloride and pilocarpine induced seizures in rats	Decreased proinflammatory cytokines, oxidative stress, and apoptosis rate of hippocampal neurons, and enhanced cell viability	[56]
Rhein	IL-1β-mediated NF-κB signaling	Pentylenetetrazole-induced seizure in mice	Delayed onset of seizure, decreased severity, duration, and frequency of seizures	[59]
Saikosaponin a	IL-1β-mediated PI3K/AKT/mTOR signaling pathway hyperactivation	Pentylenetetrazole-Induced seizure in rats	Reduced seizure severity and duration, elevated seizure latency	[57]
Phyllathin	IL-1β-mediated NF-κB signaling	Pentylenetetrazole-induced seizure in mice	Delayed onset of seizure, decreased severity, duration, and frequency of seizures	[60]
Chondroitin Sulfate	IL-1β-mediated NF-κB signaling	Pentylenetetrazole induced epilepsy and pilocarpine induced status epilepticus in mice	Reduced seizure severity and frequency	[61]
ANXA1-derived peptide Ac2-26	Modulation of the levels of formyl peptide receptor 2 and phosphorylated extracellular signal-regulated kinase	Pilocarpine-induced status epilepticus in rats	Reduced neuronal injury and inflammation related to status epilepticus	[62]
Biochanin A	NLRP3 inflammasome, IL-1β-mediated PI3K/AKT/mTOR signaling pathway hyperactivation	Pentylenetetrazole-induced seizure in mice	Reduced seizure severity and duration	[58]

**Table 3:** Substances targeting HMGB1/TLR4 pathway in epilepsy. This table summarizes compounds that ameliorate epileptic phenotypes in animal models by inhibiting the HMGB1/TLR4 axis. Mechanisms include blocking HMGB1 translocation (e.g., Celecoxib), directly antagonizing HMGB1 or TLR4 (e.g., anti-HMGB1 antibody, TAK-242, Glycyrrhizin), and downstream suppression of Proinflammatory signaling (e.g., Fisetin, Resveratrol). Outcomes demonstrate reduced seizure severity, frequency, and associated neuroinflammation.

Substance	Targeted Mechanism	Model	Outcome	Ref.
Celecoxib	HMGB1 translocation	Lipopolysaccharides and pilocarpine induced seizures in rats	Reduced Racine score and delayed latency to generalized tonic–clonic seizures onset	[89]
		Kainic acid induced recurrent seizures in immature rats	Reduced recurrent seizures	[90]
TAK-242	HMGB1/TLR4/IκK/κBα/NF-κB signaling	Pilocarpine-induced status epilepticus in mice	Alleviates hippocampal neuronal injury, reduces levels of proinflammatory cytokines	[91]
Anti-HMGB1 monoclonal	HMGB1	Pilocarpine and methylscopolamine induced acute seizures in mice	Prolonged onset and latency of the Racine stage five	[86]
		Pentylenetetrazole-induced seizure in zebrafish	Prolonged onset of seizure and attenuated memory impairment	[87]
Fisetin	HMGB1-mediated AkT/mTOR pathway and NF-κB signaling	Pentylenetetrazole induced seizure in mice	Increased the latency for myoclonic jerks and generalized seizures	[92]
Glycyrrhizin	HMGB1	Lithium–pilocarpine-induced status epilepticus in rats	Decreased mortality and hippocampal neuronal damage	[86]
Resveratrol	HMGB1/TLR4 signaling	Pentylenetetrazole induced seizure in mice	Prolonged onset and latency of seizure	[93]

## Data Availability

No new data were created or analyzed in this study.
